# An Overview of the Antimicrobial Properties of Lignocellulosic Materials

**DOI:** 10.3390/molecules26061749

**Published:** 2021-03-20

**Authors:** Flávia C. M. Lobo, Albina R. Franco, Emanuel M. Fernandes, Rui L. Reis

**Affiliations:** 1 3B’s Research Group, I3Bs—Research Institute on Biomaterials, Biodegradables and Biomimetics of University of Minho, Headquarters of the European Institute of Excellence on Tissue Engineering and Regenerative Medicine, AvePark—Parque de Ciência e Tecnologia, Zona Industrial da Gandra, 4805-017 Barco/Guimarães, Portugal; flavialobo@i3bs.uminho.pt (F.C.M.L.); albina.franco@i3bs.uminho.pt (A.R.F.); rgreis@i3bs.uminho.pt (R.L.R.); 2 ICVS/3B’s—PT Government Associate Laboratory, 4805-017 Braga/Guimarães, Portugal

**Keywords:** lignocellulosic materials, natural fibers, bacteria, fungi

## Abstract

Pathogenic microbes are a major source of health and environmental problems, mostly due to their easy proliferation on most surfaces. Currently, new classes of antimicrobial agents are under development to prevent microbial adhesion and biofilm formation. However, they are mostly from synthetic origin and present several disadvantages. The use of natural biopolymers such as cellulose, hemicellulose, and lignin, derived from lignocellulosic materials as antimicrobial agents has a promising potential. Lignocellulosic materials are one of the most abundant natural materials from renewable sources, and they present attractive characteristics, such as low density and biodegradability, are low-cost, high availability, and environmentally friendly. This review aims to provide new insights into the current usage and potential of lignocellulosic materials (biopolymer and fibers) as antimicrobial materials, highlighting their future application as a novel drug-free antimicrobial polymer.

## 1. Introduction

The presence of pathogenic microorganisms on the material surfaces can lead to significant healthcare and environmental problems. In recent times, different strategies have been defined to prevent the proliferation and adhesion of microorganisms on medical devices; or materials for food storage, and packaging [1,2]. Moreover, the biofilm formation on the materials surfaces can limit their functionality, leading to critical health related complications [3]. Furthermore, antibiotic-resistant microorganisms have emerged due to the extensive use of antibiotics or biocidal to impair their growth. Thus, it is necessary the development of new drug free materials that could avoid the increase of antibiotic-resistant microorganisms.

**Figure 1:**
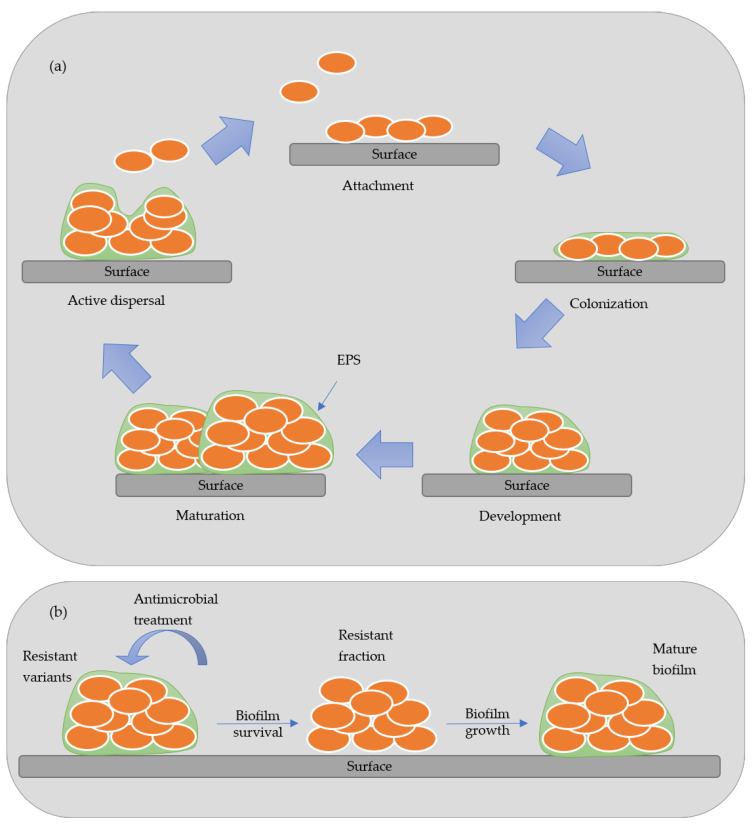
Model of biofilm formation (**a**) and antimicrobial resistant variants (**b**).

Biofilm is an aggregate of microorganisms that attaches to wet surfaces and multiplies, forming a slimy matrix of extracellular polymeric substances (EPS), thus creating an optimum environment to develop biofilms [4]. The EPS is composed of polysaccharides, proteins, lipids, and nucleic acids, forming a highly hydrated polar mixture that contributes to the three-dimensional structure of the biofilm. The biofilm formation is established in five stages: attachment, colonization, development, maturation, and active dispersal. Figure 1 presents a scheme of the stages of development of biofilm. In the attachment stage, the microorganisms are reversibly absorbed to the biotic or abiotic surface by weak van der Waals forces bonds. In contrast, in the colonization stage, stronger hydrophilic/hydrophobic bonds are established with the surfaces allowing them to proliferated and secret EPS [4,5]. In the maturation stage, a three-dimensional structure contains channels that distribute nutrients and signal molecules in the biofilm. In the last stage, called active dispersion, the cells are detached, either singly or in clumps, and colonize other locations [5]. The formation and development of biofilm depend on many factors, such as the specific bacteria strain, the properties of the material’s surface, the environmental condition (pH, temperature, and nutrients), among others [6]. Biofilms are responsible for biocorrosion, biofouling (accumulating microorganisms in surfaces), and reservoir souring, causing many constraints in different industries [4,7].

The main mechanisms of antimicrobial action by which antimicrobial compounds affect microorganism are protein synthesis inhibition, cell wall disruption, and nucleic acid inhibition. Antimicrobial compounds can act as suppressing protein synthesis targeting the ribosomal subunits or protein folding, thus inhibiting their active role. They can also disrupt cell walls, causing an increase of permeability of the membrane, leading to the leakage of intracellular constituents. In addition, they are able to inhibit nucleic acid mechanism by suppressing the replication of deoxyribonucleic acid (DNA) and ribonucleic acid (RNA) [8]. Currently, there is no deep knowledge on the mechanism of action against pathogenic microorganisms and the way they will act on surfaces.

The conventional methods to disinfect surfaces uses antimicrobial reagents, such as antibiotics, fungicides, antiviral drugs, and nonpharmaceutical chemicals [2]. Antifouling agents are also employed since they prevent the adsorption on the surface and/or kill/inhibit the growth of microbes, preventing the biofilm formation. Antimicrobial or antibacterial agents are classified as a subclass of antifouling agents, and these materials present biocidal activity [9].

The extensive use of these compounds can cause concern due to their environmental pollution potential, and the development of microbial resistance. The use of antimicrobial agents can be limited, and they cannot achieve high and durable local concentrations on the surface and providing lessen disinfection of the materials surfaces [2]. Therefore, it is important to develop new antimicrobial agents able to prevent microbe’s adhesion and proliferation on materials surfaces, and reduce their negative effects.

The antimicrobial agents are classified into two categories, organic and inorganic. The organic antimicrobial agents include natural biopolymers, for instance, the chitosan, cellulose and lignin, phenols, halogenated compounds, and quaternary ammonium salts [10,11,12]. The inorganic antimicrobial agents comprise, for example, metals, or metals bonded with phosphates, and metal oxides. The most common metallic nanoparticles or metal oxides used are silver, copper, titanium oxide, zinc oxide, magnesium, and calcium oxide [10,11,12]. In the literature, several studies explore the use of different antimicrobial agents by incorporation or applied as coatings on materials surfaces. Among them, the antimicrobial potential of natural derived lignocellulosic compounds remains still unexplored.

Since lignocellulosic materials are mainly composed by the biopolymer with antimicrobial activity, cellulose and lignin, these materials have revealed antimicrobial potential. The present review explores the use of the lignocellulosic compounds, cellulose, hemicellulose and lignin, and lignocellulosic fibers as antimicrobial agents, highlighting their antimicrobial potential to be applied for different technological applications from the environment to the health.

## 2. Lignocellulosic Materials and Main Compounds

Lignocellulosic materials are mainly composed of three biopolymers—cellulose, hemicellulose, and lignin—combined with smaller other components. The ratios between these compounds vary depending on the lignocellulosic material origin [13,14,15,16].

Recently, the antimicrobial activity of lignocellulosic materials has been explored. In Table 1, we present examples of the use of lignocellulosic materials main compounds as antimicrobial materials.

### 2.1. Cellulose

Cellulose is the most abundant renewable polymer found in nature, and it is the main constituent of the cell wall. It can be biosynthesized by different organisms, such as plants, amoebae, sea animals, bacteria, and fungi [17].

**Figure 2:**
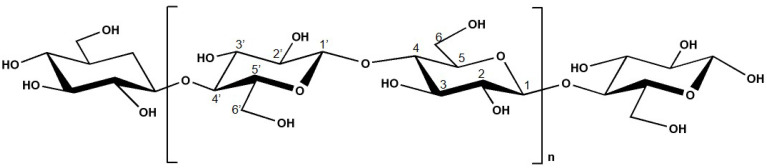
Chemical structure of cellulose.

Cellulose is a linear homopolysaccharide with the molecular formula (C_6_H_10_O_5_)_n,_ and it is composed of ß-D-glucopyranose (glucose) moieties linked by β-(1,4) glycosidic bonds [13,14,18,19]. The chemical structure of cellulose is presented in Figure 2. This compound possesses both well-ordered (crystalline) and disordered (amorphous) regions [13,14,18,20]. The presence of polar oxygen and hydrogen atoms in cellulose allows the formation of intermolecular and intra-molecular bonding [21].

The structure of this material is organized as microfibrils, with a diameter between 2 and 20 nm, connect together to form cellulose fibers [17,22]. 

Cellulose is commonly used as a raw material in different industries, such as textile, plastic, wood, cosmetics, and pharmaceutical. Cellulose presents high biocompatibility, biodegradability, non-toxicity, and high hydrophilicity. It also reveals good mechanical properties, thermal and chemical stability, chirality and allows chemical modification [23,24,25]. Cellulose can be applied in a wide range of applications, such as packaging [26,27], biomedical [28,29,30], tissue engineering [31], wound dressing [31,32,33], marine coatings [34], among others. For instance, Onofrei et al. [29], developed films composed of cellulose acetate blended with hydroxypropylcellulose. The film with a higher concentration of cellulose acetate inhibited the growth of *Escherichia coli* (*E. coli*) and *Staphylococcus aureus* (*S. aureus*).

In work by Sun et al. [27], cellulose-based membranes were produced with cellulose fibers modified by azidation, followed by epoxidation and grafted with poly(hexamethylene guanidine) (PHMB). The antibacterial activity of the membranes against *E. coli* and *S. aureus* was investigated after each modification step. Here, membranes with PHMB grafting showed higher antibacterial activity. Furthermore, the antibacterial effect lasted up to 60 days, possibly due to the covalent connection between cellulose and PHMB. It is proposed that the membranes can be used for packaging applications.

Gogoi et al. [34], also modified nanofibrillar cellulose from *Colocasia esculenta* with triethanolamine and silver (Ag) nanoparticles to produce epoxy resin composites. The combination of nanofibrillar cellulose with Ag nanoparticles presented improved antibacterial activity against *S. aureus* and antifungal activity against *Candida albicans* (*C. albicans*). Guna et al. [28], prepared cellulose fibers from the tulsi stalk and tested the antimicrobial activity against *S. aureus*, *E. coli*, *Ser. marcescens*, and *B. cereus*. The work shows a bacteria reduction between 55% and 62% for the nanofibrillar cellulose, and a higher reduction of 90% to 98% for the tulsi stalk fiber. The same trend was also observed by Ilangovan et al. [32], where fibers made from cellulose extracted from *Curcuma Longa L*. residues. Gabov et al. [35], showed that beads prepared from the combination of cellulose and lignin obtained from birch wood chips presented antimicrobial activity against *S. aureus*. However, the beads had a high concentration of lignin in the matrix, which could have influenced the antibacterial activity. Yadav et al. [31], prepared bio-sponges from a composite of sodium alginate and cellulose extracted from mango wood scrap combined with bio-extracts from rice water and Giloy extract. The bio-sponges demonstrated good antibacterial activity against Gram-positive, *B. subtilis*, and Gram-negative bacteria, *E. coli*, and *P. aeruginosa*. Oliva et al. [26], isolated the cellulose from paper with concentrated sulfuric acid to produce films that were then treated with zinc oxide and carvacrol essential oil. The films made with cellulose extract showed good antibacterial activity against *E. coli* and *S. aureus*, but the ones made with the essential oil only presented a considerable reduction of the bacteria at higher concentrations (2 wt.%). On the other hand, hydrogels made with cellulose obtained from sugarcane bagasse and combined with zinc oxide nanoparticles present good antimicrobial activity against *S. aureus* (bacteria) and *T. rubrum* (fungi). It was verified that the zinc nanoparticles enhanced the already existing antimicrobial properties of the cellulose. Furthermore, Anagha et al. [37], suggested that the good antimicrobial properties allied with their excellent biocompatibility and low cytotoxic, making these hydrogels good for biomedical applications. Likewise, films made with nanocellulose obtained from oil palm or empty fruit bunches via alkaline treatment and acid hydrolysis combined with zinc oxide showed good antibacterial activity against *E. coli* and *S. aureus* higher than the activity observed for the zinc oxide particles [52]. Overall, these studies indicate that cellulose derived from different sources demonstrates good antimicrobial activity, thus inferring to the potential of these materials.

The antimicrobial activity of cellulose can be enhanced by the inclusion of inorganic nanoparticles. Li et al. [30], prepared composites by combining titanium dioxide and cellulose that presented higher inhibitory activity against *E. coli* than *S. aureus*. On the other hand, Li et al. [53] also prepared composites with silver chloride and cellulose that demonstrated good antibacterial activity against both *E*. *coli* and S. *aureus*. Furthermore, other studies developed composites made with cellulose, silver nanoparticles, and other commercial polymers such as Polyvinyl alcohol (PVA) [54] and polyurethane (PU) [55], also exhibit good antibacterial properties, suggesting that the addition of commercial polymers have no additional effect on the antibacterial properties of cellulose-based composite.

### 2.2. Hemicellulose

**Figure 3:**
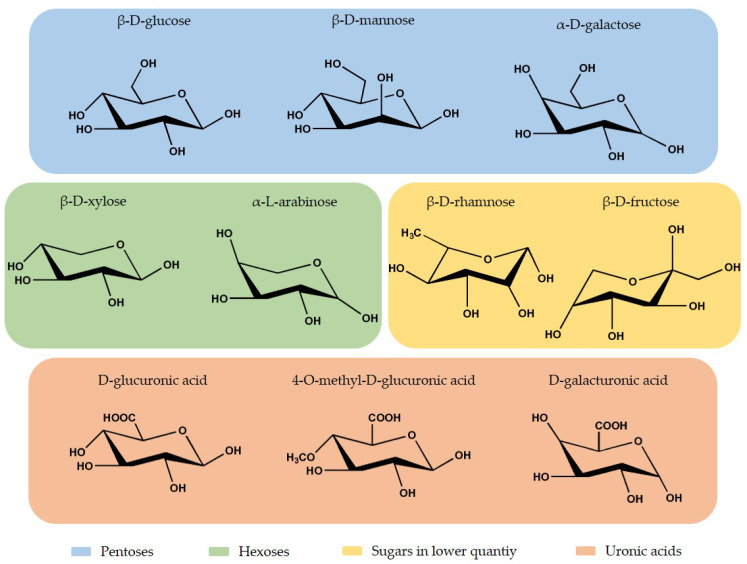
Chemical structure of hemicellulose monosaccharides units. Adapted from Wang [58], with permission. Copyrigth 2017, Elsevier.

Hemicellulose is a short-chain heteropolysaccharide present in the cell wall, and it corresponds to 15% to 35% of the plant composition, depending on the plant species [56,57,58]. The heteropolymer presents an amorphous branched structure and a lower polymerization degree than cellulose, approximately 200 [58]. Hemicellulose is constituted by different monosaccharide units, hexoses (β-D-glucose, β-D-mannose, and β-D-galactose) and pentoses (β-D-xylose and α-L-arabinose) in higher quantities, and other sugars (fructose and rhamnose) in lower quantities. It also presents uronic acids, like 4-O-methyl-D-glucuronic acid, D-glucuronic acid, D-galacturonic acid, and acetyl groups [19,56,58,59,60]. In Figure 3, is possible to observe the chemical structure of the monosaccharides units present in hemicellulose. 

Thus far, the antimicrobial properties of hemicellulose are less explored when compared to cellulose and lignin. Nevertheless, these polymers offer great antimicrobial potential due to their components. Ahmad et al. [38], the hemicellulose films inhibited the growth of the bacteria *S. aureus*, *E. coli*, and *P. aeruginosa*. Bouaziz et al. [39], the authors assessed the antibacterial activity of hemicellulose extracted from almond gum against different strains of Gram-positive (*B. subtilis*, *B. thuringiensis*, *Actinomyces* sp., *L. monocytogenes*, and *S. aureus*) and Gram-negative bacteria (*P. aeruginosa*, *Sal. enterica*, *Salmonella typhimurium*, and *K. pneumoniae*). Here, the hemicellulose presented higher inhibitory activity against *B. thuringiensis*, *S. enterica*, and *P. aeruginosa* and moderated inhibition against *Actinomyces* sp., *Sal*. *thyphimirium*, *K. pneumonia*, *L. monocytogenes*, and *B. subtilis*. However, the hemicellulose antibacterial activity was lower when compared to the positive control. Nevertheless, the work suggests that hemicellulose has a bacteriostatic behavior against the tested bacterial, showing that these materials could be used for food or non-food applications. 

The antimicrobial activity of xylan is one of the most studied polymers derived from hemicellulose extract. Fu et al. [61], showed that gels using xylan, gelatin glycerol, and Nicotinamide had good antimicrobial activity against Yeast but lower activity against *B. subtilis* and *S. aureus.* Moreover, the gels displayed good cytocompatibility to be applied in cosmetic industries. Arellano-Sandoval et al. [62], also produced hydrogels with xylan extracted from bagasse and Poly(N-vinylcaprolactam) that inhibited the bacterial growth of *E. coli*, *S. aureus*, and *P. aeruginosa*. 

### 2.3. Lignin

Since 1977, when Adler [63] described lignin as a highly branched polymer with different functional groups: aliphatic and phenolic hydroxyls, carboxylic, carbonyl, and methoxyl groups, the structure of lignin has significantly been studied and explored. Lignin is one of the most abundant polymers in nature, and it is an amorphous heterogeneous polymer network of phenylpropane units linked together by different, non-regular sequence bonds [13,14,64].

**Figure 4:**
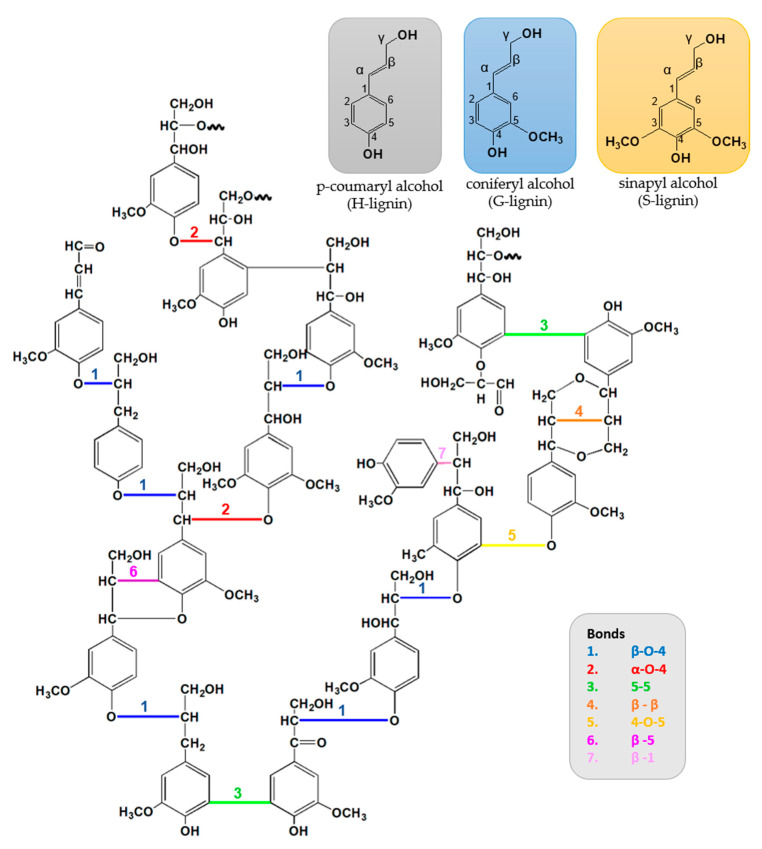
Chemical structure of monolignols, softwood lignin, and the type bonding formed between the monolignols during the polymerization process. Adapted from Windeisen [72] with permission. Copyright 2012, Elsevier.

The phenylpropane units are originated from three aromatic alcohol precursors, monolignols, p-coumaryl (H-lignin), coniferyl (G-lignin), and sinapyl (S-lignin) alcohols. During the polymerization process, the monolignols units are linked by radical coupling reactions to form the three-dimensional molecular architecture with different bonds, and typically around half of these bonds are β-4-O ether connections [65,66,67,68,69]. Figure 4 presents the chemical structure of lignin with the highlighted bonds between the monolignols.

Lignin behavior is similar to a thermoplastic, presenting decomposition temperature and glass transition temperature that varies with the isolation method, absorbed water, molecular weight, and thermal history [65].

This compound is responsible for binding the lignocellulosic materials’ components, acting as a glue, making it insoluble in water. Lignin presents an essential role in woody plants; it is the major component of the vascular plant’s cell wall and confers rigidity, impermeability, resistance to microbial attack, and oxidative stress. Lignin is mainly used to generate heat and energy but is also used as a food and concrete additive, dispersants, resin, and binding material [13,14,18,64,70].

The lignin’s antimicrobial activity is influenced by different factors, such as the origin or the extraction method. Several lignin’s extracted with different methods are described in the literature, such as kraft lignin, hydrolysis lignin, organosolv lignin [71]. In the kraft process, the lignocellulosic materials are treated with a solution of sodium hydroxide (NaOH) and sodium hydrosulphide (NaHS) in a temperature range of 150 to 170 °C. After the treatment, the lignin ether bonds are cleaved, and lignin is converted to small fragments, also known as alkali-soluble lignin. For the organosolv method, lignin is dissolved in organic solvents (acetic acid, ketone, and ester), and the organosolv lignin is highly pure, sulphuric-free, and has less modification.

To understand the effect of the origin and the extraction method and how the lignin origin influence in antimicrobial properties, Gordobil et al. [41], used lignin obtained from eucalyptus and spruce by two different methods, kraft, and organosolv. The author used solutions with different lignin concentrations to evaluate antifungal activity against *A. Niger* and antibacterial activity against different bacteria (Table 1). It was concluded that the kraft lignin’s present higher antifungal activity than the organosolv lignins. In the kraft lignin from eucalyptus, presents a high antifungal activity for all the concentrations, however spruce lignin only acts as antifungal in lower concentrations. In this work, it is proposed that the higher antifungal activity observed in the kraft lignins could be explained by the lower carbohydrate content and by the presence of sulfur-containing derivatives. For the antibacterial tests, the authors verified that the kraft lignins present higher antibacterial activity, in some cases, higher than the commonly used antibiotic, due to their rich antioxidant and polyphenolic nature. This lignin’s revealed potential to be applied as antimicrobial additive or agent against pathogenic microorganisms in food, textile and chemical industries. As shown in a previous study, antifungal activity is not only influenced by the extraction method but also by the origin. The results obtained by García et al. [44], show that the organosolv lignin extracted from apple tree pruning residues could not show the antifungal activity against *A. niger*. However, the authors could demonstrate the resistance against other fungi, *Saccharomyces cerevisiae*.

Another promising method to extract lignin from natural fiber is the use of ionic liquids. Ionic liquids are green solvents with high thermal stability and present low toxicity [73]. In the work by Shen et al. [42], lignin extracted from poplar wood was combined with epichlorohydrin (ECH) and polyethylene glycol (PEG) to produce membranes. The lignin powder’s antibacterial activity extracted by kraft method and ionic liquid (1-ethyl-3-methylimidazolium acetate) method and the membranes were tested against *E. coli*. The kraft lignin powder presented 56% of bacteria reduction, while ionic liquid extracted lignin only presented 26% of bacteria reduction. The membranes produced with both types of lignin also showed a reduction of the bacteria on the surface. The developed membranes could be used for drug delivery, food packaging, and wound dressing.

Some authors use the fractionation method to extract lignin. Fractionation is a physical-chemical modification technique that allows the separation of high molecular weight lignin chains from lower molecular weight fractions [74]. In the work by Wang et al. [49] used enzymatic hydrolyzed lignin from corn stalk and performed two sequential ethanol extraction to obtain a different lignin fraction. The authors tested the antimicrobial activity of the lignin extracts against the Gram-positive bacteria, *E. coli and B. subtilis*, and the Gram-negative, *Sal. enterica* and *S. aureus*. The authors observed that the first extract showed higher antibacterial activity against all the bacteria, but the Gram-positive are more sensitive to the lignin extract than the Gram-negative bacteria. Likewise, they also extracted lignin from bamboo by the kraft method, and fractionated with ethanol to obtain a soluble and an insoluble fraction [51]. The kraft lignin and the lignin fractions antimicrobial activity was tested against the same bacteria used in the previous study. The insoluble phase showed low inhibition against Gram-positive bacteria. It promoted the growth of the Gram-negative bacteria probably by the low phenolic compounds content and poor water solubility, leading to the formation of insoluble particles that act as carriers for the bacteria. The soluble phase showed good growth inhibition of the growth of both types of bacteria [51]. Kaur et al. [46], modified lignin from sugarcane bagasse by three different methods, acetylation, epoxidation, and hydroxymethylation and, evaluated the antibacterial activity against the bacteria *B. aryabhattai* and *Klebsiella* sp. The author verified that lignins antibacterial activity depends on the concentration, and lignins are more effective against *B. aryabhattai* than *Klebsiella* sp. In the modified lignins, the minimum inhibitory concentration (MIC) value of the epoxy lignin is the lowest, and the acetylated lignin is the highest for both bacteria. Despite the promising results, the unmodified and modified lignin present lower antibacterial activity than standard tetracycline. The authors indicate that the modified lignins could be used as a natural antimicrobial agent.

Lignin can also be functionalized with metallic nanoparticles to improve antibacterial and antifungal activity. In the work by Aadil et al. [75], present lignin from acacia was functionalized with silver nanoparticles that displayed antibacterial efficacy against Gram-positive and Gram-negative strains. Chandna et al. [76], functionalized kraft lignin with gold (Au) and silver (Ag) nanoparticles. The bimetallic nanoparticle, composed of Au and Ag, showed better antibacterial and antifungal activity than the nanoparticle systems composed by lignin and Au or lignin and Ag.

Lignin composites have also demonstrated that the addition of lignin promotes antimicrobial activity. In the literature, it is shown the combination of different polymers such as with poly(butylene succinate) (PBS) [40], alginate [43], and poly(vinyl alcohol) (PVA) [77]. The composites obtained by Domínguez-Robles et al. [40], by the extrusion followed by injection molding of kraft lignin extracted from softwood with PBS, showed higher antimicrobial activity against *S. aureus* than the PBS matrix, even in a small amount (2.5 wt.%). Aadil et al. [43], also showed the antibacterial effect of lignin against *S. aureus*. The authors extracted lignin from Acacia wood powder and produced composite films with alginate. Lignin composites presented antibacterial activity against *S. aureus*, although the composites did not show any effect against *E. coli*. Lee et al. [77] used alkali lignin combined with PVA to produce fiber by electrospinning process. The composites with 50% and 85% of lignin presented 99.9% of reduction of *S. aureus*, but no effect was observed for *E. coli* bacteria.

In this context, the materials produced with lignin can be considered for different areas of application, such as biomedical [40,50,78], textile [47,50], packaging [42,43] and as natural antimicrobial agent [46,49,51].

## 3. Lignocellulosic Fibers

**Figure 5:**
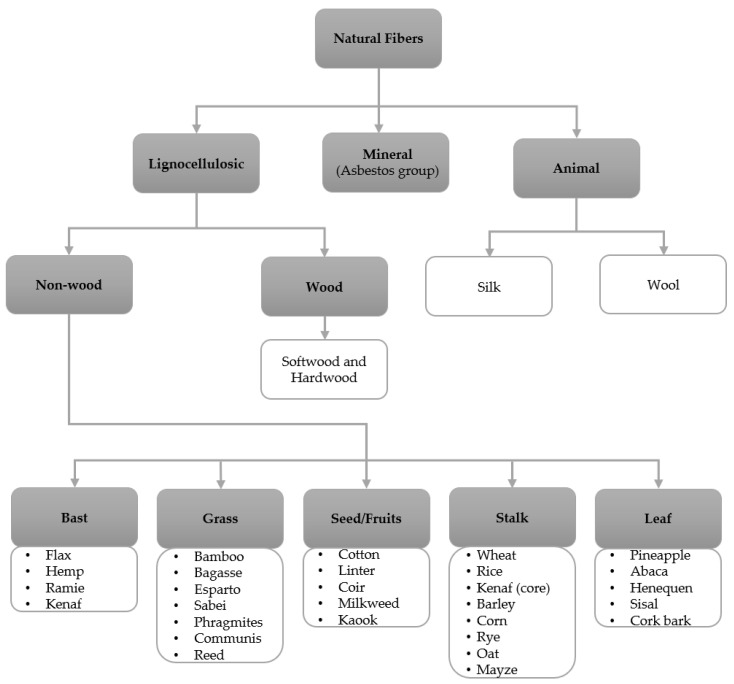
Hao [79], and Gurunathan [81], with permission. Copyright 2018 and 2017, Elsevier.

Natural fibers are mainly classified into three different classes in accordance with the origin of the fiber: plant, animal, and mineral [79,80], as shown in Figure 5, and the main chemical composition of each fiber in Table 2. The lignocellulosic fibers are mainly classified in bast, grass, seed/fruit, leaf or hard fibers, stalk, and wood (hardwood and softwood) [81]. The plant fibers are also classified as primary and secondary plants. The primary plants are cultivated for their fiber, such as jute or hemp, and the secondary plants are grown for the fruit, but the by-products of the plants are used to produce fibers, for example, banana and pineapple [80]. 

The lignocellulosic fibers present unique properties, such as low specific weight, high specific strength, good mechanical properties, good thermal, and acoustic insulation properties. These materials from renewable sources are environmentally friendly, high availability, low cost, biodegradability, low amount of energy during fiber processing, contributing to a lower emission of carbon dioxide, and they do not produce harmful gases [79,80,81,82,83,84,85]. As the main drawbacks, the natural fibers present high hydrophilicity, which causes high moisture absorption, poor matrix-fiber interfacial adhesion, and low fiber dispersion when combined with polymer matrices [80,85]. However, this can be overcome with the fiber surface modification or the use of coupling agents [79].

Traditionally, natural fibers are used to produce ropes, fabrics, cords, and threads. Engineering applications can be broader since they can be used in the automotive, packaging, paper, marine, and aerospace industries [79,80].

### 3.1. Wood Fibers

As previously described, the origin of the lignocellulosic material influences the antibacterial and antifungal activity. Munir et al. [91], described the evaluation of the antibacterial activity of wood disks from European fir (*Abies alba*), American red oak (*Quercus rubra*), European oak (*Quercus spp*.), and European beech (*Fagus sylvatica*). From them, the European oak species showed positive activities against a Gram-negative bacteria, *P. aeruginosa*, and a Gram-positive bacteria *E. faecalis*. 

In polypropylene composites with wood flour, the origin of the wood flour was also observed. The antifungal activity of wood flour from Chinese white poplar (*Populus tomentosa*), moso-bamboo (*Phyllostachys heterocycla*), Chinese fir (*Cunninghamia lanceolata*), Ramin (*Gonystylus bancanus*), Chinese white pine (*Pinus armandii*), river red gum (*Eucalyptus camaldulensis*), western red cedar (*Thuja plicata*), and rubberwood (*Hevea brasiliensis*) was tested against *A. niger*, *Trichoderma viride*, *Penicillium funiculosum*, *Aureobasidium pullulans*, and *Chaetomium globosum* [92]. The authors concluded that the mold growth resistance depended on the wood origin, with Chinese fir, red gum, and red cedar showing better activity against the fungi tested. They also established a relationship between the lower sugar content of wood fiber rendered WPC with higher fungal resistance, suggesting that composite from wood with less sugar content presented higher antifungal activity.

The use of wood flour in polymer composites with antimicrobial activity is reported in the literature for different polymer matrices, such as polyvinyl chloride (PVC) [93], polyhydroxyalkanoate (PHA) [94,95], polylactic acid (PLA) [96], poly(3-hydroxybutyrate-co-3-hydroxyvalerate) (PHVB) [97] and polyvinyl alcohol (PVA) [98]. The composites with PHA and PLA presented good antibacterial activity against *E. coli*. The PHA composites with a higher quantity of wood (20% or more) in the composition exhibit more antibacterial activity [94]. In the case of PLA composites [96], the author used triclosan, an antibacterial material in different concentrations. The authors observe that PLA/triclosan composites showed lower antibacterial activity than the composites with PLA/wood flour/triclosan against *E. coli*, proving that the wood flour increases the antibacterial activity. 

Not only the origin of the material influence the antimicrobial activity; so too does the type of fiber. Treinyte et al. [98] prepared composites with PVA with materials from the same tree, pine, but they used pine needles and pine bark. The composite with pine needles did not present antifungal activity, but the composites with pine bark partially suppressed *Trichoderma viridescens* and did not affect the other fungus. Overall, the pine materials did not present good antimicrobial activity.

The combination of wood material with non-wood materials also revealed promising results. Jamili et al. [93] produced PVC composites with wood and wood dyed with walnut shell wood by extrusion process. The composites showed a reasonable reduction of the growth of *S. aureus* and *E. coli*; however, the composites dyed with walnut shells proved to be the most antibacterial due to the presence of phenolic and naphthoquinone compounds in walnut.

### 3.2. Non-Wood Fibers 

Different studies propose non-wood fibers as antimicrobials agents. These fibers can be presented as found in nature, or with different treatments, or combined with polymers. Kalinoski et al. [99] studied different hydrogels prepared with poplar wood and sorghum dissolved with ionic liquids. They compared the antimicrobial activity against *E. coli* of the lignocellulosic hydrogels with hydrogels prepared with commercial lignin, xylan, and cellulose. They verified that the hydrogels compositions based on cellulose/lignin, cellulose/lignin/xylan, and poplar presented a significant reduction of *E. coli*.

The work by Gonçalves et al. [100] shows that cork has antibacterial activity against *S. aureus*, demonstrating that after 90 min of incubation, the reduction of the bacteria was about 100%. This behaviour is similar to the value obtained for a commercial product known to inhibit bacteria growth. The cork behaviour in the presence of *E. coli*, showed a bacterial reduction of only 36%. These results are explained by the differences in the bacteria’s cell wall, and the Gram-negative bacteria present an outer membrane that acts as a barrier. Francesko et al. [101], functionalized the cork particles with silver nanoparticles produced in the presence of chitosan or 6-deoxy-6-(ω-aminoethyl) aminocellulose. The work reveals that only the functionalization of cork particles with silver nanoparticles increased the antibacterial activity against *E. coli* and *S. aureus*.

In order to increase the antimicrobial activity of lignocellulosic fibers, some authors treated fibers with other compounds. Ketema et al. [102] proved that the cotton fibers treated with nettle leaf extract present antibacterial properties against *E. coli* and *S. aureus*. Li et al. [103], removed the lignin and hemicellulose from the hemp fibers and impregnated them with a Cinnamon derivative. The authors concluded that the fibers treated with cinnamon derivatives also have higher antimicrobial activity against brown-rot and white-rot fungi. 

In the work by Thakur et al. [104], coconut fiber also showed antimicrobial activity. The authors modified the coconut fibers by biografting the lignin structure with ferric acid and proved that they have antibacterial activity against *E. coli* than *S. aureus*. Lazić et al. [105] prepared flax fibers with different hemicellulose content and lignin and combined them with Ag nanoparticles. The samples with a lower lignin concentration present higher antimicrobial activity against *E. coli*, *S. aureus*, and fungi *C. albicans*.

The incorporation of different lignocellulosic materials in PBAT/starch composites was studied by Spiridon et al. [106]. The composites were prepared with celery fibers, poplar seed hair fibers, pomace, and *Asclepias syriaca* fibers. The pomace composite was the only material that presented inhibition against both bacteria *E. coli* and *S. aureus*. Torres-Giner et al. [107] prepared composites with poly(3-hydroxybutyrate-co-3-hydroxyvalerate) (PHBV) and coconut fibers and coconut fibers impregnated with essential oregano oil by extrusion process. The films with the functionalized coconut fiber, presented antibacterial activity even for a low concentration of the fibers. Guna et al. [90] prepared composites with sabai fiber and polypropylene (PP). The sabai fibers presented higher antibacterial activity against *S. aureus* and *B. cereus* compared to *E. coli* and *S. marcescens* and better inhibition against the fungus *Cryptococcus* than *A. niger*.

## 4. Intellectual Property

Patent rights are designed to confer only a market opportunity. Furthermore, they create the opportunity for patent owners to obtain higher returns for products or services of the claimed technical solution [108]. There is a particular increasing interest in materials demonstrating efficient use of renewable resources, that is reflected by the increasing number of publications during the recent years. A search carried out in Espacenet Patent Search Database (Jan 2021) revealed a total of 84 applications related to strategies containing lignocellulosic materials and claiming antibacterial properties. The survey revealed that the vast majority of the patented technologies are based on different application areas, whereas nine patent cases on the wood-polymer composite (WPC) materials. Table 3 reports the recent patents and technologies that have shown us a wider application in lignocellulosic antibacterial materials. As expected in this area, the majority of antibacterial properties of several lignocellulosic materials are achieved by adding the inorganic or organic agents or incorporating the antibacterial agent on a coating that is further applied in the lignocellulosic material.

WPC are a group of innovative materials consisting of mainly renewable resources. Typically, the concept is based on the selection of waste materials and by-products from wood and agricultural industry as raw materials that are combined with thermoplastic or thermosetting matrices and a small amount of additives that are further processed by melt-based technologies to obtain the desired product. Furthermore, with the increase in the variety and content of filled lignocellulose, the fast development of the industry, and the continuous expansion of the application fields, the resistance of WPC to biotic and abiotic factors has decreased significantly. Thus, there are some effective innovations claiming antibacterial resistance, such as the invention CN104893331A [109], that pretreats the lignocellulosic powder by spraying a chitosan-nanosilver composite as antibacterial agent on the wood powder surface. After that, the material is mixed and combined during the processing with a thermoplastic such as polyolefins. The resulted WPC material has favorable resistance to *S. aureus*, *E. coli*, *P. aeruginosa*, being a safe and environmentally friendly product solution. In this area, it is evident that the metal oxide particles have an effective antimicrobial activity against common harmful microorganisms and have been used to pre-treat the lignocellulosic fraction before compounding. For instance, the CN106752049A [110], reports the use of titanium dioxide (TiO_2_), the invention CN101659751A [111], includes the addition of zinc oxide (ZnO) or the invention CN108841188A [112], reports a WPC material that uses carbon nanofibers to enhance thermal conductivity, and the antibacterial agent is a silver-zinc composite. 

A different concept can be found in the embodiment CN105350741A [113], where the WPC flooring system comprises on the wear surface a decorative layer based on polyvinyl chloride (PVC) that contains the antibacterial agent, and it is applied through a rolling method. According to the claims, the antibacterial coating is a photocatalyst layer that is also applied in the CN106183293A [114]. Similarly, the innovation CN109731747A [115], discloses the use of a nano-oxide suspension by indicating a layer of polydimethylsiloxane that is sprayed on the surface of the WPC and further dried to prepare an anti-corrosion antibacterial composite material. The nano-oxide suspension is nano-aluminum oxide, a nano titanium dioxide, and/or a nano zinc oxide suspension, resulting in a claimed antiseptic, antibacterial wood fiber composite material.

Besides, the innovation reports the technology to prepare an antibacterial WPC by using sawdust, peanut shell, rice husk, several crop straws, linen rods, and cotton rods. The lignocellulosic raw material is crushed and pulverized into powder and dried and further combined with thermosetting or a thermoplastic by using an extrusion process. In the composition, it is also used other additives that may confer the claimed property.

Table 3 also presents other strategies and uses claiming the antibacterial property. Among the patents, the EP2199046A1 [117], describes the use of organic compounds, in this case, oligomeric or polymeric tannins that are covalently bonded onto the surface of wood or other lignocellulosic materials by enzymatically catalyzed oxidation. In this regard, the modified lignocellulosic surface shows improved antibacterial (bactericidal or bacteriostatic) properties compared to the untreated surfaces. In the innovation CN108724381A [118], a technology for wood floor based on an antibacterial impregnation process is proposed that comprises a vapor treatment chamber to use reduced amounts of the antibacterial agent. 

Additional patents focus on the potential of the chemical constituents of the lignocellulosic materials. The invention CN105506765A [119], reports a method for produce a functional regenerated cellulose fiber. In particular, the invention comprises the steps of dissolving a cellulose pulp, introducing a material containing a graphene structure and non-carbon non-oxygen elements (e.g., Fe, Si and Al), and obtaining a spinning dope. The functional regenerated cellulose fiber has far-infrared antibacterial properties, bacteria resistance, and bacteriostasis with relevance for clothing, home textiles, or special protective clothing for industrial use.

Lignin has also been considered to develop polyurethane products, as reported in the invention CN105637036A [120]. Polyurethanes foams are typically formed by the reaction of a resin comprising at least one polyol, a surfactant, a catalyst and a blowing agent, and the isocyanate comprising two or more isocyanate groups. The polyols used are usually derived from petroleum products. Nevertheless, due to environmental concerns, the industry is now attempting to replace petroleum products with bio-based solutions, for instance, derived from biomass, such as agricultural waste or biomass in forests. In the invention, an antibacterial agent is added to the composition, the lignin is less expensive and less harmful to the environment than the traditional petro-based polyols.

Lignocellulosic materials are considered non-toxic, odorless, non-pollution, and non-radioactive and can be used as a food packaging film. However, cellulose molecules contain a large number of hydroxyl groups, which tend to form intermolecular and intramolecular hydrogen bonds that increases crystallinity. The invention CN107934198A [121], presents the use of ellagic acid for modifying lignocellulose to reduce the effect of hydrogen bonding and crystallinity changes on the crystal structure of cellulose. The remarkable result is that the thermoplastic processing performance of cellulose is greatly improved, and at the same time, the thermal stability performance is ensured as well as the barrier function of the biopolymer film targeting packaging application. 

Looking at intellectual property (IP) in the same database and combining the words cellulose or lignin with antibacterial property, we obtain thousands of IP results. One area of particular interest due to the social impact and high added-value of the products is the biomedical field sector. In this regard, hydrogels are a swelling body that has a three-dimensional polymer network structure, formed by physical crosslinking or chemical crosslinking, containing a large amount of water but insoluble in water [122]. The range of benefits includes softness, rich moisture content and good biocompatibility to be used in biomedical, tissue engineering, sensors, among others. The invention CN110240774A [123], discloses a polyvinyl alcohol (PVA) that contains a large amount of hydroxyl groups formed by physical or chemical crosslinking to form a hydrogel. The use of lignin in the mixture with a solvent reinforces the PVA, resulting in hydrogels by freeze–thaw method or solvent exchange method. The hydrogels show a high-strength lignin/polyvinyl alcohol composite with good electrical conductivity and antibacterial activity. 

A different use in the invention AU2007236166A1 [124], relates to biomedical foam articles to treat chronic wounds, which are formed by spraying a polymer onto a wound surface to form a three-dimensional spatial shape, covering the wound surface and is also highly absorbent. The most frequent forms of chronic wounds by far are decubitus ulcers (caused by chronic pressure), chronic venous ulcers of the legs (caused by chronic venous insufficiency) and diabetic ulcers (caused by angiopathy and neuropathy). The standard treatment of chronic wounds follows the principle of “moist wound healing” with different wound contact materials. In this particular case biomedical foam composition uses naturally ionic biopolymers based on carbohydrates such as cellulose derivatives, for example cellulose acetate phthalate, cellulose acetate succinate, cellulose acetate trimellitate, hydroxypropylmethylcellulose phthalate, carboxymethylcellulose, and also natural biopolymers such as lignin contributing to the wound healing process. Furthermore, in the field of biomedical materials, there are inventions taking advantage of the strength and antibacterial properties of bamboo fiber. The patent CN106075601A [125], discloses a bamboo fiber that is prepared as a porous material as a reinforcing phase of hydroxyapatite/polylactic acid composite material, or in the patent CN108607116A [126], that presents a method to combine bamboo fiber with nano-apatite, where both inventions claim application in bone tissue engineering scaffold materials. 

The IP in this field showed that depending on the natural lignocellulose material innovation, it can be applied in outdoors, or indoor houses and finishing, hospitals, antibacterial package, biomedical and other places that have high requirement on the anti-aging, mildewproof and antibacterial performances of the material.

## 5. Conclusions and Future Perspectives

Lignocellulosic materials are widely used in several production sectors such as construction, furniture, packaging, or the automotive industry. Several studies have highlighted the potential of these natural fibers or their chemical constituents on different polymer-matrix systems. In addition, their antimicrobial effects have been recognized. In the last few years, the use of lignocellulosic materials has grown, mainly to their characteristics; high availability, environmentally friendly, from renewable sources, low cost, and biodegradability. This review presents an overview of the most recent advances that demonstrates the potential of the lignocellulosic-based materials, cellulose, hemicellulose, lignin, and lignocellulosic fibers to be used as antimicrobial agents. In this area, the antimicrobial activity of the materials has emerged from the combination of the lignocellulosic source with antimicrobial agents from inorganic or organic origin. The intellectual and industrial property similarly shows products following the same routes; however, there are several innovations in the field that claims antibacterial activity only because at least one of the constituents present in the material is known to have an antibacterial effect. Thus, further research efforts in respect of these findings are needed, preferably in the presence of certain bacteria or fungi showing the inhibition of bacterial growth.

The potential of lignocellulosic as new drug-free polymers is extensive, but this area is still virtually unexplored, especially as antimicrobial or anti-biofouling materials for industries, such as healthcare, environmental, textile, space engineering, among others. By unlocking the full potential of the antimicrobial properties of lignocellulosic materials, it will be possible to fully disclose their potential, bringing new links of knowledge between the areas involving the synthesis of natural fibers, polymer matrices, and microbiology.

## Figures and Tables

**Table 1:** Antimicrobial activity of lignocellulosic compounds.

Compound	Origin	Antimicrobial Activity Tested Against	Application	Ref
Cellulose	Wood	*E. coli*, *S. aureus*	Packaging	[27]
		*E. coli*, *S. aureus*		[35]
		*E. coli*, *P. aeruginosa*, *B. subtilis*	Tissue engineering, wound dressing	[31]
	Sugarcane Bagasse	*S.aureus*, *T. rubrum*	Skin infective	[36]
			Wastewater purification	[37]
	Tulsi	*E. coli*, *S. aureus*, *B. cereus*, *Ser. marcescens*	Biomedical	[28]
	Ginger	*E. coli*, *S. aureus*, *B.cereus*, *Sal. thyphimirium*	Packaging, wound dressing, surgical material	[33]
Hemicellulose	Plantago Ovata seed husk	*E. coli*, *S. aureus*, *P. aeruginosa*	Wound dressing, drug delivery	[38]
	Almond gum	*Actinomycetes sp*, *Sal. thyphimirium*, *K. pneumonia*, *L. monocytogenes*, *S. aureus*, *Sal. enterica*, *P. aeruginosa*, *B. thuringiensis*, *B. subtilis*	Food and non-food	[39]
Lignin	Softwood	*S. aureus*	Biomedical	[40]
	Eucalyptus	*A. niger* *E. coli*, *S. aureus*, *Pr. microbilis*, *Pr. vulgaris*, *P. aeruginosa*, *Entero. aerogenes*, *B. thuringiensis*, *Sal. enterica serotype typhmurium and Strept. mutans*	Antimicrobial additive or agent in food, textile, or chemical industry	[41]
	Spruce			[41]
	Poplar	*E. coli*	Drug delivery, food packaging, wound dressing,	[42]
	Acacia	*E. coli*, *S. aureus*	Active packaging	[43]
	Apple tree pruning residues	*A. niger*, *Sacch. cerevisiae*	Food antioxidant	[44]
	Sugarcane Bagasse	*E. coli*, *S. aureus*, *P. aeruginosa*, *S. epidermidis*		[45]
		*B. aryabhattai*, *Klebsiella* sp.	Natural antibacterial agent	[46]
		*S. epidermidis*	Antimicrobial textile	[47]
	Corn	*L. monocytogenes*, *S. aureus*, *E. coli*, *Sal. enteritidis*, *C. lipolytica*	Antioxidant and antimicrobial	[48]
		*E. coli*, *S. aureus*, *B. subtilis*, *Sal. enterica*	Natural antibacterial agent	[49]
	Cotton stalks	*S. aureus*, *K. pneumoniae*	Medical and technical textiles	[50]
	Bamboo	*E. coli*, *S. aureus*, *B. subtilis*, *Sal. enterica*	Natural antibacterial agent	[51]

Legend: *Aspergillus niger* (*A. niger*); *Bacillus aryabhattai* (*B. aryabhattai*); *Bacillus cereus* (*B. cereus*); *Bacillus subtilis* (*B. subtilis*); *Bacillus thuringiensis* (*B. thuringiensis*); *Candida lipolytica* (*C. lipolytica*); *Enterobacter aerogenes*, (*Entero. aerogenes*); *Escherichia coli* (*E. coli*); *Klebsiella pneumoniae* (*K. pneumoniae*); *Listeria monocytogenes* (*L. monocytogenes*); *Proteus microbilis* (*Pr. microbilis*); *Proteus vulgaris* (*Pr. vulgaris*); *Pseudomonas aeruginosa* (*P. aeruginosa*); *Saccharomyces cerevisiae* (*Sacch. cerevisiae*); *Salmonella enteritidis* (*Sal. enteritidis*); *Salmonella enterica* (*Sal. enterica*); *Salmonella thyphimirium* (*Sal. thyphimirium*); *Serratia marcescens* (*Ser. marcescens*); *Staphylococcus aureus* (*S. aureus*); *Staphylococcus epidermidis* (*S. epidermidis*); *Streptococcus mutans* (*Strept. mutans*); *Trichophyton rubrum* (*T. rubrum*).

**Table 2:** Lignocellulosic fibers composition.

	Fiber	Cellulose (%)	Hemicellulose (%)	Lignin (%)	Ref
**Wood**	Softwood (Pine)	45.0–50.0	25.0–35.0	25.0–35.0	[86]
	Hardwood (Poplar)	50.8–53.3	26.2–28.7	15.5–16.3	[86]
**Non-wood**	Apple tree pruning	75.81	7.84	4.03	[44]
	Bamboo	30.60	17.00	3.41	[51]
	Cork	6–25		13–26	[87]
	Cotton	82.7–92	5.7–6	0	[88]
	Flax	71–81	18.6–20.6	2.2–3	[88]
	Hemp	70.2–74.4	17.9–22.4	3.7–5.7	[88]
	Pineapple	70–82	15–19	5–12	[88]
	Sabai Grass	42.9	21.1	18.5	[89]
	Sisal	56.5–78	5.6–16.5	8–14	[88]
	Sugarcane Bagasse	42.11	28.42	19.29	[90]

**Table 3:** Results of patent search on antimicrobial lignocellulosic-related materials.

Publication Number	Title	Priority Year	Ref
**Wood-polymer composites (WPC)**			
CN104893331A	Antibacterial wood–plastic composite and preparation method thereof	2015	[109]
CN106752049A	Novel antibacterial wood–plastic board and preparation method thereof	2016	[110]
CN101659751A	Natural lignocellulose material with modified zinc oxide and preparation method thereof	2009	[111]
CN108841188A	Wood–plastic composite material for enhancing heat conductivity with carbon nanofiber and preparation method thereof	2018	[112]
CN105350741A	Wood–plastic flooring and manufacturing method therefor	2015	[113]
CN106183293A	Wood–plastic floor	2016	[114]
CN109731747A	Preparation method for anticorrosive antibacterial wood fiber composite	2018	[115]
CN108789762A	Preparation technology for antibacterial environmentally friendly wood–plastic composite material	2018	[116]
**Other uses claiming antibacterial property**			
EP2199046A1	Lignocellulosic substrates with enhanced antibacterial properties and method for obtaining those	2008	[117]
CN108724381A	Antibacterial impregnation treatment equipment for wooden floor and process thereof	2018	[118]
CN105506765A	Functional regenerated cellulose fiber and preparation method and application thereof	2015	[119]
CN105637036A	Process for the preparation of lignin based polyurethane products	2013	[120]
CN107934198A	Lignocellulose-ellagic acid bio-plastic food packaging film and preparation method	2017	[121]
